# Colorectal carcinomas with mucinous differentiation are associated with high frequent mutation of *KRAS* or *BRAF* mutations, irrespective of quantity of mucinous component

**DOI:** 10.1186/s12885-020-06913-2

**Published:** 2020-05-08

**Authors:** Xiaodong Li, Katherine Sun, Xiaoyan Liao, Haijuan Gao, Hongfa Zhu, Ruliang Xu

**Affiliations:** 1 Department of Pathology, NYU Langone Medical Center, New York, NY USA; 2 Present address: Department of Pathology, University of California Irvine, Orange, CA USA; 3 Department of Pathology, University of Rochester Medical Center, Rochester, NY USA; 4 Department of Pathology, Mount Sinai Medical Center, New York, NY USA; 5 Department of Pathology, White Plains Hospital, Montefiore Health System, White Plains, NY USA

**Keywords:** Mucinous adenocarcinoma, Colorectal carcinoma, Mucinous differentiation, *KRAS*, *BRAF*, EGFR signaling pathway

## Abstract

**Background:**

Mucinous adenocarcinoma (MAC) is a distinct type of colorectal cancer (CRC) associated with poor response to treatment and poorer prognosis. MAC is diagnosed by WHO definition when the extracellular mucin is more than 50% of the lesion. We aimed at assessing the gene expression profiles of the CRCs with any mucinous features (> 5%) in a retrospective study.

**Methods:**

The data of a 50-gene next generation sequencing (NGS) panel of 166 CRCs was analyzed and the gene mutational profile with morphologic features was correlated.

**Results:**

We identified the different genetic mutation profiles between CRCs with and without mucinous component, but noticed a similar genetic profile between MACs and CRCs with mucinous component, irrespective of the percentage (if mucinous component more than 5%). The different genetic mutation profile related to MSI status was also identified between two groups of tumors. The most frequent mutations in CRCs with mucinous component are *KRAS* (28/49, 57.1%) and *BRAF* (19/49, 38.7%), *PIK3CA* (16/49, 32.6%), followed by *APC* (12/49, 24.5%) and *TP53* (11/49, 22.5%). The combined mutation frequency of the two key factors in the EGFR signaling pathway, *KRAS* and *BRAF*, in the CRCs with and without mucinous component is 95.9 and 52.1%, respectively.

**Conclusions:**

The dysregulation of EGFR pathway plays a critical role in the development of CRCs with mucinous component, irrespective of the percentage. The result suggested that the current cut off of 50% mucin component to define mucinous adenocarcinoma might be challengeable.

## Background

Colorectal cancer (CRC) is the third most common cancer and the fourth leading cause of cancer-related death worldwide [1, 2]. Mucinous adenocarcinoma (MAC) is a unique histologic variant of CRC characterized by the extracellular deposition of mucin by the tumor cells. This tumor usually occurs in the right colon, more in females, and has a poorer response to adjuvant chemotherapy and chemoradiotherapy [3–5]. The WHO classification arbitrarily defines MAC as tumors with more than 50% of mucinous component, and all studies have been performed to describe the clinicopathologic and molecular features of this type of tumor based upon this definition. With the development of new technology, particularly the next generation sequencing (NGS), the MACs should be redefined by their molecular profile to more accurately predict their clinical outcomes and particular responsiveness to targeted therapy.

Anti-epithelial growth factor receptor (EGFR) has been approved to be a targeted therapy for metastatic colorectal cancer (mCRCs) since 2009, but only a small percentage of mCRC patients shown sensitivity to this therapy, largely due to *KRAS or BRAF* mutations leading to constitutive activation of EGFR signaling pathway [6, 7]. *KRAS* mutations are present in approximately 35 to 40% and *BRAF p. V600E* in 14% of colon cancers with nearly mutually exclusive genotype between each other [6]. The mutation of these two genes is frequently seen in MACs [8–10], resulting in a poor response to anti-EGFR therapy [7, 11, 12]. However, approximately 35% of conventional CRCs are also positive for *KRAS* and *BRAF* mutations. So far, very little studies have studied whether therapeutic efficacy of anti-EGFR therapy can be attributed to mutation of *KRAS* and *BRAF* associated with mucinous differentiation (less than 50%), and whether a different amount of the mucinous component could affect the genetic expression.

In this retrospective study, we analyzed the data of a 50-gene NGS panel of 166 CRCs and correlated the gene mutational profile with morphologic features in a set of 49 CRCs with varying amounts of mucinous component. The aim of this study is to identify a distinct molecular genetic associated with the mucinous differentiation, providing molecular basis for the clinical outcome and biological behavior in this subset of tumors.

## Methods

### Case selection and histology review

We selected 166 patients with CRC who underwent surgical resection without neoadjuvant treatment and with NGS testing and MSI data at NYU Langone Medical Center and Mount Sinai Hospital from July1, 2016 to June 30, 2017. The corresponding pathology reports were reviewed for demographic information including age and sex. The slides were reviewed by three GI pathologists to confirm histopathology, including tumor grade, stage, lymph node metastasis, immunostain for mismatch repair proteins, and mucinous differentiation, based upon the 8th edition of AJCC staging manual. The tumors were extensively (at least one section per centimeter of tumor) examined to evaluate the percentage of extracellular mucin associated with malignant epithelium. The overall concordance rate of diagnostic interpretations of mucin presence percentage among the three participating GI pathologists is 85%. For those discrepant cases, a consensus score was generated after re-examination of the slides. The cases with more than 5% but less than 50% of mucinous component were classified as CRCs with mucinous feature. The rest of CRCs without mucin or less than 5% of mucinous component were classified as conventional CRCs. The study was approved by the Institutional Review Board of the New York University.

### Next-generation sequencing

One representative section of a tumor with mucinous component confirmed by three GI pathologists was chosen for NGS testing. Genotyping results were taken from the accompanying Ion AmpliSeq™ Cancer Hotspot Panel v2 reports. The gene panel tested 207 regions covering approximately 2800 mutations from 50 oncogenes and tumor suppressor genes. The panel includes *ABL1, AKT1, ALK, APC, ATM, BRAF, CDH1, CDKN2A, CSF1R, CTNNB1, EGFR, ERBB2, ERBB4, EZH2, FBXW7, FGFR1, FGFR2, FGFR3, FLT3, GNA11, GNAS, GNAQ, HNF1A, HRAS, IDH1, IDH2, JAK2, JAK3, KDR, KIT, KRAS, MET, MLH1, MPL, NOTCH1, NPM1, NRAS, PDGFRA, PIK3CA, PTEN, PTPN11, RB1, RET, SMAD4, SMARCB1, SMO, SRC, STK11, TP53, VHL*.

### Genotype-phenotype correlation

To investigate the correlation of percentage of mucinous components to genetic profile, CRCs with mucinous feature were divided into three groups based upon the percentage of mucinous component: < 30% but > 5%, between 30 and 50%, and ≥ 50%. The 30% category was designed to show the genetic expressions of tumors with a small percentage of mucinous features, which usually missed when reviewed by the general pathologist according to the WHO diagnostic guide. The gene mutations detected by NGS among three groups were analyzed and compared. The conventional CRCs were divided into two groups: cancer with ≥3 gene mutations and cancer with < 3 gene mutations. Tumor grade, T stage, lymph node metastasis, and MSI status in the two groups were compared. Both CRCs with and without mucinous feature were further divided into MMR- proficient and MMR- deficient groups based upon immunostain for mismatch repair proteins (MLH1, MSH2, MSH6 and PMS2). The genetic profiles of those cancers were investigated.

### Statistical analysis

Chi squared (X^2^) and Fischer’s Exact (FE) tests were applied when appropriate. Two-tailed, with *p*-values < 0.05 was designated as statistically significant.

## Results

### Clinicopathologic features

A total of 166 cases of CRCs met the inclusion criteria for this study (Table 1). Briefly, female/male ratio is 1.30:1 and median age is 64 years (ranging from 32 to 91). The tumors were located in the cecum (38 or 22.9%), the ascending colon (34 or 20.5%), the transverse colon (18 or 10.8%), the descending colon (8 or 5.0%), the sigmoid colon (38 or 22.9%) and the rectum (30 or 18.0%). Across the entire cohort, 7 (4.2%) of them were classified as pathological stage T1, 10 (6.0%) as T2, 97 (58.4%) as T3, and 52 (31.3%) as T4. Immunohistochemical study for mismatch repair proteins (MLH1, MSH2, MSH6 and PMS-2) as surrogate markers for microsatellite instability was performed as part of routine assessment, and 42 (25.3%) of the cases were found to be MMR- deficient by immunohistochemistry. CRCs without mucinous component made up 70.5% of the cohort (*n* = 117). 85 of 117 (72.6%) were morphologically classified as low grade (well and moderately differentiated) and the remaining 32 (27.4%) as high grade (poorly differentiated). At least 5% mucin by tumor volume was identified in 49 of 166 cases (29.5%). Among them, 16 cases (32.7%) had less than 30% mucinous component, 9 cases (18.4%) had 30–50% mucinous component, and 24 cases (49.0%) had more than 50% mucinous component. No follow-up and survival data were available due to short period of time after surgical resections.

**Table 1:** The association between mutation genes number and clinicopathologic features

	Total	< 3 mutations (*n* = 97)	≥ 3 mutations (*n* = 69)	*P* value
*Gender*				*p* > 0.05
*Male*	72	44	28	
*Female*	94	53	41	
*Age*	32–91	33–91	32–88	
*Location*				*p* > 0.05
*Cecum*	38	14	24	
*Ascending colon*	34	19	15	
*Transverse colon*	18	11	7	
*Descending*	8	4	4	
*Sigmoid colon*	38	29	9	
*Rectum*	30	20	10	
*Histologic grade*				***p*** **= 0.0010**
*Low*	126	82	43	
*High*	40	15	26	
*Pathologic stage*				*p* > 0.05
*T1*	7	4	3	
*T2*	10	6	4	
*T3*	97	57	40	
*T4*	52	30	22	
*Positive lymph nodes*				*p* > 0.05
*Yes*	99	62	37	
*No*	67	35	32	
*MSI status*				*P* > 0.05
*MSI-proficient*	124	76	48	
*MSI-deficient*	42	21	21	
*CRC without mucinous differentiation*	117	72	45	***P*** **< 0.01**
*Low grade*	85	59	26	
*High grade*	32	13	19	

### Frequency of gene mutations across colorectal carcinomas

The majority of CRCs (155 of 166, 93.4%) were found to have at least 1 mutation detected by NGS. The most commonly mutated gene was *TP53* (77, 46.4%), followed by *KRAS* (76, 45.8%), *APC* (54, 32.5%), *PIK3CA* (43, 25.9%), *BRAF* (33, 19.9%), *SMAD4* (18, 10.8%), *FBXW7* (15, 9.0%) and *PTEN* (14, 8.4%). Mutations seldom associated with CRCs were *KIT, GNAS, JAK2, RB1, RET, FGFR2, NOTCH, PIPN11, ERBB2, IDH2, ABL1,* and *KDR*. In addition, multiple concurrent mutations were found to be common in our cohort. Two concurrent mutations were found in 61 CRCs (36.7%), 3 in 40 cases (24.1%), 4 in 23 cases (13.9%), 5 in 5 cases (3.0%), and 6 in 1 case (< 1%).

### Molecular and pathologic characteristics of CRCs with mucinous differentiation

The most commonly mutated genes in CRCs with at least 5% mucinous component were *KRAS* (28/49), *BRAF* (19/49), *PIK3CA* (16/49), followed by *APC* (12/49) and *TP53* (11/49). In concordance with the results of study done by Gonsalves et al. [6], our study has shown that *KRAS* and *BRAF* mutations are almost mutually exclusive and only one case had both mutations in this group. The combination mutation rate of either *KRAS* or *BRAF* in this group of CRCs is 95.9% (47/49). Neither *KRAS* nor *BRAF* was identified in 2 of 49 cases this group. *TP53* mutation was identified in one of these two tumors and the mutation of *AKT1*, *APC*, and *PTEN* were identified in other case. In the group of CRCs without mucin (or mucin component < 5%), 61 of 117 cases had either *KRAS* (48/117, 41.0%) or *BRAF* (13/117, 11.1%) mutation, and no double mutations. The combination mutation rate of either *KRAS* or *BRAF* is 52.1% (61/117). *TP53* mutation (66/117) was the most frequent mutation in this group, followed by *KRAS* (48/117, *APC* (42/117), *PIK3CA* (27/117) and *BRAF* (13/117).

**Fig. 1:**
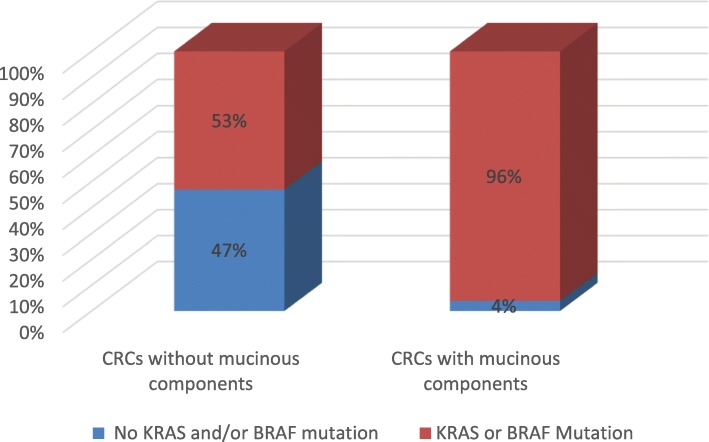
Dysregulation of EGFR Pathway in Carcinoma with and without Mucinous component

These results showed that tumors with any amount of mucin (more than 5%) had a significantly higher likelihood arising from the serrated pathway as evidenced by *BRAF* mutation (38.7%, compared with 11.1% among conventional CRCs, *p* < 0.001). Seventeen *BRAF* mutations detected were *BRAF p. V600E* (class 1) and other *BRAF* mutations were *BRAF p. W604L* (unclassified) and *BRAF p. G469A* (class 2) [13]. All *KRAS* mutations detected in tumor with mucinous differentiation were within codon 12 (21 cases), 13 (3 cases), 61 (2 cases), 117 (1 case) and 146 (1 case). *NRAS* mutations were not identified in this study. Combined *KRAS* mutation, the EGFR signaling pathway in this group is near completely blocked due to either *KRAS* or *BRAF* mutation (Fig. 1, *p* < 0.001). Our data also showed that the mutation distribution among three subgroups of CRCs with mucinous features (< 30% mucin, 30–50% mucin, and > 50% mucin) did not have significant difference, suggesting that they share the same or similar clinically relevant molecular genetics or biology (Table 2). Since the tumor with either *KRAS* or *BRAF* mutation has a poor response to anti-EGFR therapy, any mucinous component (more than 5%) may be an adverse indicator for poor responsiveness to anti-EGFR therapy.

**Table 2:** The association between percentage of mucinous components and *KRAS/BRAF* mutation

% of mucinous components (*n* = 49)	*KRAS* mutation (*n* = 28)	*BRAF* mutation (*n* = 17)	Both gene mutation (n = 1)	Nether gene mutation (*n* = 3)
*< 30% (n = 16)*	7	7	0	2
*30–50% (n = 9)*	6	3	0	0
≥ *50% (n = 24)*	16	7	1	0

Total 42 tumors were defined as MMR- deficient based on the results of immunochemical stain. 23 tumors had > 5% mucinous component (23/49, 46.9%) and 19 tumors had no mucinous feature (19/117, 16.2%) (*p* < 0.0001). In both groups of CRCs with and without mucinous component, *BRAF* mutation was frequently identified in MMR- deficient tumors (15/23 vs. 4/26 and 6/19 vs. 7/98, *p* = 0.001), while *TP53* was more common in MMR- proficient tumors (10/26 vs. 1/23 and 61/98 vs. 519). However, *KRAS* mutation was frequently identified in MMR- proficient tumors with mucinous components (20/26 vs.8/23), while *PTEN* mutation was more common in MMR- deficient tumors with mucinous components (6/23 vs. 2/26). These findings were not shown in conventional tumors (Table 3).

**Table 3:** The association between genes mutation with MSI status of CRC with and without mucinous components

Gene mutation	CRC with mucinous components			CRC without mucinous components		
	MMR-deficient (*n* = 23)	MMR-proficient (*n* = 26)		MMR-deficient (*n* = 19)	MMR-proficient (*n* = 98)	
*KRAS*	*8*	*20*	*p = 0.001*	*8*	*40*	
*BRAF*	15	4	*p = 0.001*	6	7	*p* = 0.007
*APC*	3	9		5	37	
*TP53*	1	10	*p* < 0.05	5	61	*p* < 0.05
*PIK3CA*	9	7		7	20	
*SMAD4*	2	7		1	8	
*PTEN*	6	2	*p* = 0.125	2	4	
*FBXW7*	2	1		5	7	

### High tumor grade is correlated with increased number of gene mutation in conventional CRCs

In 117 conventional CRCs, 72 (61.5%) had < 3 gene mutations and 45 (38.5%) had ≥3 gene mutations. In cancers with < 3 gene mutations, 59 (81.9%) were low grade (well and moderately differentiated) and 13 were (18.1%) high grade (poorly differentiated) CRCs. In the cancers with ≥3 gene mutations, 26(57.8%) were diagnosed as low and 19(42.2%) as high-grade CRCs. The number of mutated genes is strongly associated with high tumor grade (*p* = 0.0010). High T stages (stage 4) does not correlate with mutation status in the cancers with < 3 genes mutations (30/97) and with more than 3 gene mutations (22/69) (*p* > 0.05). In addition, no correlation between the numbers of gene mutations and positive lymph nodes or MSI status was found in the cancers with < 3 gene mutations (62/97 or 21/97) and with more gene mutations (37/69 or 21/69) (*p* > 0.05). Since the grading of MAC is controversial and not recommended by WHO, the tumors with > 5% of mucinous components were exclude when correlating tumor grade and the number of genes mutation.

## Discussion

This study demonstrated that the CRCs with mucinous component, irrespective of the proportion of mucin volume (more than 5%), have similar clinically relevant molecular genetics (i.e., *KRAS* and *BRAF* mutations), and their genetics are different from that of CRCs without mucinous feature. The findings suggest that CRCs with mucinous feature and MAC may belong to the same group with unfavorable biological behavior or resistance to anti-EGFR treatment. Our results point out the disadvantage to classify the mucinous CRSs without the molecular data, suggesting that the current classification does not necessarily serve clinical needs in the era of precision medicine.

MAC is a distinct type of CRCs, arbitrarily defined by more than 50% composition of extracellular mucin. This definition was first described by Parham in 1923 [3] and adopted by WHO in 1989 ever since. This variant of tumor occurs in 1.6–25.4% of all CRCs [5, 10], and bears poor prognosis and resistance to treatment. According to a meta-analysis, MACs result in 2–8% increased hazard of death, which persists after correction for stage [5], and this tumor also showed a poorer response to both adjuvant chemotherapy and chemoradiotherapy [3, 4]. The difference of the biological and clinical behaviors between the conventional CRCs and MACs has a molecular basis, for example, increased microsatellite instability (MSI) [14], CpG island methylation phenotype [15] and, *PIK3CA* [10], *TGFR* [10] and *BRAF* mutation in MACs [6, 8, 10]. However, the conventional CRCs comprise of tumors with variable amount of mucin (0–49%), and they may have variable genetics. It is unclear if the genetics and biology of those CRCs with more than 5% mucinous component is closer to MACs or conventional CRCs. This leads us to investigate this group of CRCs with mucinous component to determine whether any amount of mucinous component is associated with similar or variable genetics or clinical outcome. Based upon NGS results, we found that CRCs with more than 5% but less than 50% of mucin share the similar clinically relevant molecular profile with CRCs to those with more than 50% of mucinous component. Similar to Fuchs’s study [16], we found that both ≥50 and < 50% mucinous tumors showed similar molecular features with more frequent *BRAF* or *KRAS* mutations in comparison to tumors without mucinous component; this distinct molecular profile has no correlation with amount of mucinous component in the tumors as long as the mucin is more than 5%. Like in other studies, these two gene (*KRAS* and *BRAF*) mutations were mutually exclusive [6, 8]. Consistent with the previous studies [17, 18], our result had the same increased proportion of cases of MMR- deficient mucinous adenocarcinoma. Furthermore, our result showed that *PTEN* mutations tended to occur more frequently in MMR-deficient tumors with mucinous components than conventional tumors (6/23 vs. 2/26), though the result is not statistically significant (*p* = 0.125 using Fisher exact). These findings indicated that genetic mutation profiles related to MSI status are different between tumors with and without mucinous components. Different from previous report, this study emphasized that almost all tumors with mucinous differentiation have either *KRAS* or *BRAF* mutations (47 of 49 cases). Thus, the histology in combination with molecular data could more accurately determine the biology of the mucinous CRCs or CRCs with mucinous feature for appropriate management.

The mutations of *KRAS* and *BRAF* are associated with poor prognosis or worse disease-free survival in adjuvant setting [19]. There are near complete blockage of EGFR signaling pathway due to either *KRAS* or *BRAF* mutation in CRCs with mucinous feature, which may have a significant clinical implication. Anti-EGFR antibodies such as cetuximab or panitumumab have become routine treatment regimens either as a single agent or in combination with conventional chemotherapy to metastatic CRCs (mCRCs) [20]. The main mechanism of resistance to anti-EGFR in CRCs is the mutation of *KRAS* (35–50% of patients) or *BRAF (*10–15% of patients) [7, 11, 12]. Our finding may explain why only a small percentage of mCRCs with mucinous feature are sensitivity to this therapy [7]. This may also provide a molecular basis for non-responsiveness in a substantial number of patients with “conventional” CRCs to this regimen because of the heterogeneity of conventional CRCs with variable amount of mucinous component less than 50%. This study showed that any amount of mucin is a poor predictor for the anti-EGFR therapy; hence the arbitrary cut off of 50% mucin component to define MAC is challengeable. During clinical practice, a small percentage (5–10%) of mucinous component is easily missed or not mentioned in the clinical pathologic report. A more objective, standardized histologic analysis together with molecular data is needed to update the classification of this MAC or CRC with mucinous feature.

In additional, we also noticed that accumulation of gene mutations identified by NGS was closely correlated with tumor histologic grade, but not tumor stage, lymph node involvement, and distant metastasis. Most of CRCs are sporadic and only up to 25% of patients have a family history of CRCs [21]. A series of molecular genetic alterations have been identified in tumorigenesis of CRCs [22], confirming the concept that sporadic CRCs result from the progressive accumulation of genetic and epigenetic alterations, leading to the transformation of normal colonic mucosa to adenocarcinoma [1]. This finding suggested that the accumulation of gene mutations results in a worse biological behavior.

Due to a limited number of cases and low sensitivity of NGS panel in this study (a limited number of genes included in the hotspot mutations detection instead of the whole gene sequencing), more detailed morphology-molecular correlation was not achieved, such as lower *APC* mutation rate in CRC and frequency of *KRAS* subtype mutations in MACs or CRCs with mucinous feature. Nevertheless, this is a study opening a venue to address the issue of classification of MACs in more objective way and identify histologic predictor for anti-EGFR therapy. A large study using more comprehensive gene panel may provide more definitive answers.

## Conclusion

In summary, we identified characteristic mutational profile associated with CRCs with mucinous feature, regardless of percentage of mucinous component, and a positive association of tumor grade with mutational accumulation. Therefore, mucinous differentiation could be an indicator of poor response to anti-EGFR therapy. A more objective, standardized histologic analysis together with molecular data is needed to update the classification of MACs and CRCs with the mucinous feature.

## Data Availability

The datasets generated and/or analyzed during the current study are not publicly available due to patients’ information protection but are available from the corresponding author on reasonable request.

## Abbreviations

MAC: Mucinous adenocarcinoma
CRC: Colorectal cancer
WHO: World health organization
AJCC: American Joint Committee on Cancer
*KRAS*: Kirsten rat sarcoma
EGFR: Epidermal growth factor receptor
*BRAF*: V-Raf murine sarcoma viral oncogene homolog B
NGS: Next generation sequencing
NYU: New York University
